# The Role and Function of microRNA in the Pathogenesis of Multiple Myeloma

**DOI:** 10.3390/cancers11111738

**Published:** 2019-11-06

**Authors:** Hiroshi Handa, Yuki Murakami, Rei Ishihara, Kei Kimura-Masuda, Yuta Masuda

**Affiliations:** 1 Department of Hematology, Gunma University Graduate School of Medicine, 3-39-22 Showa-machi, Maebashi, Gunma 371-8511, Japan; 2 Department of Laboratory Science, Gunma University Graduate School of Health Sciences, 3-39-22 Showa-machi, Maebashi, Gunma 371-8511, Japan; m13203034@gunma-u.ac.jp (Y.M.); m13203005@gunma-u.ac.jp (R.I.); m14711024@gunma-u.ac.jp (K.K.-M.); yuuken.0420@gmail.com (Y.M.)

**Keywords:** microRNA, multiple myeloma, epigenetics, drug resistance, exosome, biomarker

## Abstract

Recently, attention has been drawn to the role of non-coding regions of the genome in cancer pathogenesis. MicroRNAs (miRNAs) are small non-coding RNAs with 19–25 bases of length that control gene expression by destroying messenger RNA or inhibiting its translation. In multiple myeloma (MM), the expression of several miRNAs, such as miR-15a and miR-16, is markedly decreased and their target genes upregulated, suggesting their role as tumor-suppressing miRNAs. In contrast, miRNAs such as miR-21 and miR-221 are highly expressed and function as oncogenes (oncomiRs). In addition, several miRNAs, such as those belonging to the miR-34 family, are transcriptional targets of p53 and mediate its tumor-suppressive functions. Many miRNAs are associated with drug resistance, and the modulation of their expression or activity might be explored to reverse it. Moreover, miRNA expression patterns in either MM cells or serum exosomes have been shown to be good prognostic markers. miRNA regulation mechanisms have not been fully elucidated. Many miRNAs are epigenetically controlled by DNA methylation and histone modification, and others regulate the expression of epigenetic modifiers, indicating that miRNA and other epigenetic effectors are part of a network. In this review, we outlined the roles of miRNAs in MM and their potential to predict MM prognosis and develop novel therapies.

## 1. Introduction

In recent years, in various diseases including cancer, attention has been focused not only on abnormalities in genes encoding proteins but also on epigenetics—the control of gene expression. Epigenetics, which includes mechanisms such as DNA methylation, histone modifications such as acetylation, methylation, phosphorylation, ubiquitination, and sumoylation, and non-coding RNA such as microRNA (miRNA), long non-coding RNA (lncRNA), small interfering RNA (siRNA), and piwi-interacting RNA (piRNA), has attracted attention as a carcinogenic mechanism that differs from chromosomal translocations and gene point mutations. Non-coding RNAs are RNAs that do not translate to proteins and have been recognized as junk, but recent studies revealed that these RNAs play an important role in regulating gene expression and functions. In multiple myeloma (MM), oncogene activation by immunoglobulin heavy chain (IgH) translocations and oncogene mutations are considered to be the most important oncogenic mechanisms; however, a number of epigenetic abnormalities have been reported and their importance is becoming clear. miRNA is thought to be one such epigenetic mechanism and many miRNA abnormalities related to pathogenesis and prognosis have been discovered in many cancers including MM. In this review, we describe the relationship between miRNA abnormalities and MM, outlining their generation and mechanism of action.

## 2. What Is miRNA?

miRNAs (or miRs) are small RNA molecules with only 19 to 25 bases in length that do not encode proteins. miRNAs were discovered in 1993, when it was found that small RNAs encoded by the lin-4 locus control the development of *Caenorhabditis elegans* by modulating the expression of lin-14 proteins [1]. The importance of this small RNA molecule was recognized in 2000, upon the discovery of a small RNA molecule named let-7, which is 21 nucleotides long and highly conserved between species, from worms to humans [2]. By forming a regulatory complex (RNA-induced silencing complex, RISC) with Argonaute proteins, miRNAs are involved in a variety of biological processes, including cell proliferation, cell death, cell lineage determination, stem cell maintenance, and temporal regulation of developmental stages, by triggering the degradation and translational repression of target messenger RNAs (mRNAs) with complementary sequences. Less than 30 years after its discovery, more than 3000 miRNAs have been identified in humans. Many miRNAs are highly conserved across evolution, although their diversity and number correlate with organismal complexity [3,4,5]. The *C. elegans* repertoire contains 437 miRNAs and the mouse repertoire over 1500, whereas humans produce between 2000 and 3000 miRNAs. The number of protein-coding genes per se is similar in higher eukaryotes and simple organisms, suggesting that this variety of miRNAs is responsible for the complexity of the organisms.

## 3. Mechanisms of miRNA Production and Action

**Figure 1:**
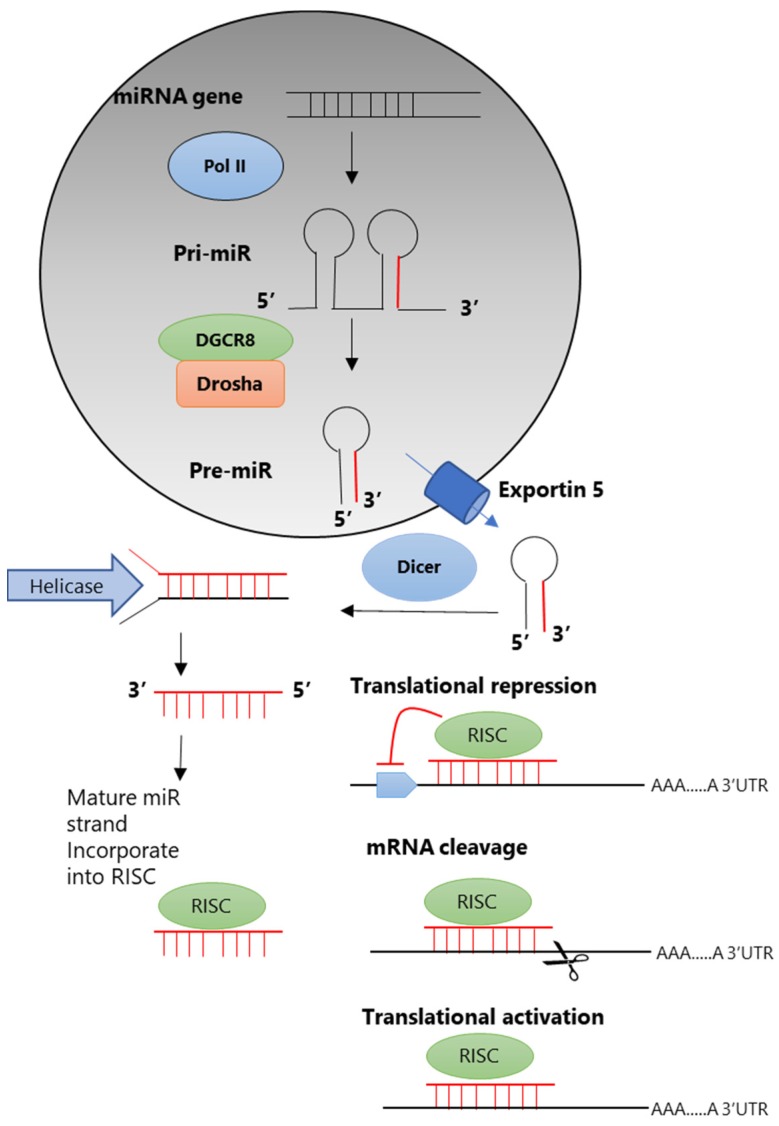
Biogenesis and mechanisms of action of MicroRNAs (miRNAs) [7]. RNA-induced silencing complex, RISC.

Most miRNAs are located in regions between genes, but recent studies have shown that they are also located within intron regions and even within protein-coding exon regions. The process of miRNA synthesis is as follows. The miRNA sequence present in the DNA is transcribed by RNA polymerase II (RNA pol II) into relatively large precursor miRNAs called primary miRNAs (pri-miRNAs). In the nucleus, pri-miRNAs are converted to precursor miRNAs (pre-miRNAs) of up to 70 nucleotides with a hairpin loop by a microprocessor complex consisting of Drosha (RNase III) and the RNA-binding protein DGCR8 (DiGeorge syndrome critical region gene 8: Pasha). Pre-miRNAs are then exported to the cytoplasm by a transporter called exportin-5, and the hairpin is cleaved into miRNAs/miRNAs* (miR-3p/miR-5p) by the RNase III family member Dicer bound to TRBP (trans-activation response RNA binding protein), PRKRA, and Argonaute (AGO 1–4). In addition, miRNAs that are unwound to single-stranded molecules by helicase are complexed with Argonaute (AGO1, AGO2) and TRBP to form miR-RISC, a complex that controls gene expression. The recognition of target mRNAs by miRNAs is mainly done by complementary base pairing of only seven bases at the 5’ end called seed sequences, and miRNAs (RISC) bound to complementary target mRNAs suppress their translation (translation of an mRNA into a protein) and degrade them [6,7,8] (Figure 1). Recent research has revealed that miRNAs reversely amplify translation under certain conditions and bind to DNA, thereby controlling gene expression at the transcriptional level, which reveals the complexity of the roles of miRNAs in the regulation of gene expression.

## 4. Cancer and miRNAs

miRNAs are frequently present in unstable regions of chromosomes that are prone to deletion and amplification in cancer cells, and it has been suggested that abnormalities in miRNAs are not limited to some cancers but are directly related to the oncogenic mechanism itself. The first finding showing a relationship between cancer and miRNA was in chronic lymphocytic leukemia (CLL). In CLL, deletions are often found in the chromosomal region 13q14, but no protein-coding gene has been found at this deletion site and the pathologic significance of the deletion has long been unclear. Calinet al. showed that there are two miRNAs in this region, miR-15a and miR-16-1 [9,10], and their expression was reduced in CLL with 13q14 deletion. Cimmino et al. also showed that this reduced miRNA expression was involved in the development of CLL because miR-15a and miR-16-1 targeted the apoptosis inhibitor BCL-2 [11]. Several other studies also revealed that miR-15a/16-1 targets multiple genes that are related to cell cycle, apoptosis, and angiogenesis such as BCL-2, MCL-1, CCND1, WNT3A, and VEGF [9]. In addition to miR-15a/16-1, many miRNAs have now been confirmed to be involved in carcinogenesis, cancer progression, and prognosis either as tumor suppressor genes or as oncogenes.

miRNAs either contribute to or repress the cancer phenotype. Many miRNAs have been discovered to be underexpressed in cancer tissues compared to their normal counterparts. Given that most of the underexpressed miRNAs in cancers can suppress tumor proliferation and induce tumor cell death via blocking oncogene expression, these miRNAs are called tumor suppressor miRNAs. In contrast, miRNAs that are overexpressed in tumors are called oncogenic miRNAs (oncomiRs). 

When oncomiRs and tumor suppressor miRNAs are inhibited and stimulated, respectively, cancer cell proliferation, metastasis, and/or survival may be significantly reduced, depending on the type of cancer and the specific miRNA being affected. Thus, miRNAs have classically been categorized as either oncogenic or tumor-suppressive [12]. However, there are conflicting reports as to whether specific miRNAs are either oncogenic or tumor-suppressive, indicating that this classification of miRNA may represent an oversimplification.

## 5. miRNA Abnormalities in MM

The first evidence of the involvement of miRNA in MM pathogenesis was presented by Al Masri et al., who demonstrated that MM cell lines and MM patients’ samples displayed a lower expression of miR-125b, miR-133a, miR-1, miR-124a, miR-15, and miR-16 than their normal counterparts [13]. Distinct expression patterns of several miRNAs between monoclonal gammopathy of undetermined significance (MGUS) and MM plasma cells and normal plasma cells also suggested a role of miRNAs in MM progression [14].

The deletion of the chromosomal region 13q14 is recognized to be highly frequent in MM as well as CLL. miR-15a and miR-16 are present in chromosome 13q [15], and their expression has been reported to be decreased in MM plasma cells compared to normal plasma cells. Furthermore, miR-15a/16 has been found to inhibit cell proliferation in vitro by impairing the expression of CDC25A and cyclin D1 and D2, targeting the apoptosis inhibitory BCL-2, and inhibiting the AKT3 and NF-kB pathways that are thought to be important contributors to MM proliferation [16]. We also found that the expression of miR-15a and miR-16-1 was clearly decreased in MM plasma cells compared to normal and MGUS plasma cells and inversely correlated with BCL-2 and cyclin E expression [17]. Considering that the in vitro function of these miRNAs is to inhibit cell proliferation, it would be appropriate to consider that the reduction of miR-15a and miR-16-1 expression is involved in the pathogenesis and progression of MM.

Higher expression of miR-21 has been reported in MM plasma cells compared to normal control cells. Several reports have suggested the oncogenic property of this miRNA: interleukin (IL)-6 induces miR-21 expression in a strictly STAT3-dependent manner, ectopic expression of miR-21 in MM cells decreases apoptosis in the absence of IL-6, and miR-21 inhibition suppresses MM cell growth [14,18,19].

miR-221 and 222 exhibit their oncogenic function by targeting tumor suppressor PTEN and pro-apoptotic PUMA associated with drug sensitivity [20,21,22]. miR-92a, which is a known hypoxia-regulated miRNA, and has been found to be upregulated in many cancers, has been proven to be related to MM progression involving the c-Jun pathway [23]. 

In vitro studies have also shown that miR-29b suppresses the expression of the anti-apoptotic gene *MCL1* and induces apoptosis in cholangiocarcinoma cells, with similar results in MM cell lines [24]. We also found that *MCL-1* mRNA expression levels were inversely correlated with miR-29a and miR-29b expression levels in various MM cell lines and patient samples, and that the introduction of miR-29 in cells decreased *MCL-1* mRNA expression. *MCL-1* appears to play an important role in anticancer drug resistance because it is overexpressed in treatment-resistant MM cells, showing potential as a therapeutic target.

## 6. Chromosomal Abnormalities and miRNA Expression in MM

The most important molecular mechanism underlying MM pathogenesis is thought to be the activation of cancer-related genes by *IgH* gene translocations. Chromosomal translocations of the *IgH* gene are present in ~40–50% of MGUS and approximately 70% of MMs, and have been speculated to be important in their development and progression. Chromosomal abnormalities are also known to affect the prognosis and molecular profile of MM and are expected to correlate with miRNA expression.

Lionetti et al. analyzed the gene expression and miRNA and DNA copy numbers in MM cell lines and plasma cells from MM patients using integrated high-resolution microarray analysis. They found that 16 miRNAs (miR-22 at 17p13.3, miR-106b and miR-25 at 7q22.1, miR-15a at 13q14.3, miR-21 at 17q23, and miR-92b at 1q22, among others) present in chromosomal regions of alleles that are often disproportionately affected by chromosomal deletions or translocations in MM presented increased or decreased expression levels compared to normal plasma cells [25]. The overexpression of miRNAs located at 1q, such as miR-1231, miR-205, miR-215, and miR-488, was correlated with chromosome 1q gain, which is observed in more than 50% of patients [25]. miR-215 directly targets murine double minute 2 (MDM2), a negative regulator of p53, and IGF-1 and IGF-1R.

Regarding chromosomal losses, monosomy and deletions of chromosome 13 are detected in up to 50% of MM cases. Some of the miRNAs located in this region, like the miR-15a/16 cluster and members of the miR-17-92 cluster (miR-17, miR19a, and miR-20a), are downregulated in primary MM samples with del13q [16,26]. miR-15a and miR-16 downregulation contributes to MM pathogenesis by promoting cell growth and neoangiogenesis in bone marrow [16]. However, Corthals et al. reported that the expression of miR-15a and miR-16 present on chromosomal region 13q14 is not associated with its copy number variation (CNV) [26,27], suggesting that the downregulation of these miRNAs does not solely depend on CNV. miR-22, encoded at the 17p locus, is significantly less expressed in those patients with the 17p deletion [28].

Gutierrez et al. reported the deregulation of miRNA expression in the different genetic subtypes of MM. They found a significantly higher expression of miR-1 and miR-133a in MM with t(14; 16), which is known as high-risk cytogenetics, downregulation of miR-135b and miR-146a in MM with t(4; 14), and target genes that are involved in the IL-1 signaling pathway [29]. Chi et al also identified specific miRNA signatures associated with the most common *IgH* translocations (t(4; 14) and t(11; 14)) and del(13q) [30]. The relationship between chromosome status and miR expression is not always consistent. A table listing miRNAs with chromosomal and expression abnormalities related to the prognosis of MM that are reported to date, as well as the chromosomal regions of miRNAs and target genes of miRNAs, is provided (Table 1).

## 7. miRNA and the p53 Pathway

p53 is a transcription factor, also called "guardian of the gene,” that monitors errors in gene replication, arrests the cell cycle when errors occur, induces the expression of repair genes, and induces cell death and the removal of abnormal cells when they cannot be repaired. In cancer cells, p53 mutates at a high rate, and the mutated p53 inhibits the action of wild-type p53, thereby inducing cancer cell proliferation and suppressing cancer cell death. The p53 mutation is less common in MM but the deletion of monoallelic *TP53* by chromosome 17p deletion has been identified as the worst prognostic factor for MM [32,33,34]. The normal p53 function is suppressed by MDM2, which is also known to be highly expressed in MM cells [35], leading to a decreased activity of p53 [36]. Reduced expression of miR-192, miR-194, and miR-215, which can inhibit MDM2 and amplify normal p53 activity, was observed in MM [37,38]; thus, the underexpression of these miRNAs is supposed to be involved in MM pathogenesis.

Ectopic miR-125a-5p reduced the expression of p53 pathway-related genes, including the expression of miR-192 and miR-194, transcriptionally regulated by p53 in MM cells, while miR-125a-5p inhibitors had the opposite effect. Lentiviral-mediated stable inhibition of miR-125a-5p expression in wild-type p53 MM cells dampened cell growth, increased apoptosis, and reduced cell migration. Inhibition of in vitro MM cell proliferation and migration was also achieved by the use of synthetic miR-125a-5p inhibitors and was potentiated by the co-expression of miR-192 or miR-194 [39]. These observations reveal that miR-125a-5p acts as an oncomiR via attenuating p53-dependent tumor suppressor networks.

MiR-125b and miR-504 are direct negative regulators of p53 as they bind to the 3’-untranslated region (UTR) of p53 mRNA, and their overexpression leads to downregulation of the endogenous level of p53, inhibiting apoptosis in human neuroblastoma and lung fibroblast cells [40,41]. Deregulation of miR-125b in MM pathogenesis and drug resistance has also been confirmed [42]. Contrary to the notion of miR-125b as oncogenic function, tumor suppressive function of miR-125b was also noticed in p53 mutated MM cells in vitro model, as well as the miR-125b-dependent upregulation of tumor suppressor miR-34a found in this model [43].

The expression of miR-34a and 34-b/c is enhanced by the binding of p53 to their promoter regions and is known to inhibit the expression of the oncogene *MYC* and cyclin-dependent phosphorylation enzyme CDK6 which promote cell proliferation [44]. In this sense, miR-34a and 34b/c function as tumor suppressor genes that mediate the p53 function [44,45]. miR-34a expression is decreased in MM cells harboring the 17p deletion/*TP53* mutation, and its promoter region is frequently methylated [46]. Others and we have shown that the promoter regions of miR-34a and 34b/c were frequently methylated in MM and that the expression of these miRNAs was increased by the demethylating agent decitabine, suggesting that p53 cannot induce the expression of these miRNAs and, consequently, cannot exert at least some of its tumor-suppressive functions through miR-34 [46,47,48,49,50].

The restoration of the susceptibility of MM cells to anticancer drugs through p53 reactivation by controlling miRNA expression might be a therapeutic strategy to explore in the future.

## 8. Epigenetic Regulation and miRNA in MM

Although miRNA expression is often suppressed in many cancers including MM, the overall underlying mechanism is not yet fully elucidated. Some are thought to be due to gene deletion as described above, but not all coding regions of miRNAs are deleted and sometimes the chromosomal deletion or CNV is not correlated with miRNA downregulation. It has recently been shown that miRNA expression is itself epigenetically regulated by methylation and histone acetylation.

miR-34a/b/c, miR-124-1, miR-194-2, miR-192, miR-203, miR-152, and miR-10b-5p have been reported to be frequently methylated in MM [50,51,52]. The target genes of these miRNAs are involved in cell survival, proliferation, and drug resistance. For example, hypermethylation of miR-34a/b/c attenuates the tumor suppressor function of p53 via targeting *BCL-2, CCND1, CCNE2, CDK4, CDK6, E2F,* and *MYC* [44]. As mentioned above, others and we have shown that the promoter regions of miR-34a and 34b/c were frequently methylated in MM and that the expression of these miRNAs was increased by the demethylating agent decitabine [46,47,48,49,50]. The repression of miR-375 by methylation is the dominant mechanism for constitutive activation of the PDPK1/RPS6KA3 signaling axis in MM [53].

In addition to DNA methylation, miRNAs are regulated by other epigenetic mechanisms such as histone modification. Silencing of histone deacetylase 4 (HDAC4) by short hairpin RNAs induced miR-29b expression through the hyperacetylation of its promoter, leading to the downregulation of miR-29b pro-survival target gene *SP1*, followed by the inhibition of MM cell survival and migration, and triggering of apoptosis and autophagy by MCL-1. Treatment with the pan-HDAC inhibitor SAHA upregulated miR-29b [54]. EZH2 is an essential component of polycomb repressive complex 2 (PRC2), which silences gene expression through methylation of lysine residues of the histone tail, and is frequently overexpressed in cancer cells. EZH2 inhibition induces the expression of miR-29b together with the downregulation of the major miR-29b pro-survival targets SP1, MCL-1, and CDK6 [55]. Histone methyltransferase MMSET, which is overexpressed in approximately 15% of MM patients mainly due to t (4; 14), binds to the miR-126* promoter and represses miR-126* by increasing H3K9 trimethylation and decreasing H3 acetylation. In turn, by repressing miR-126*, MMSET induces the expression of its target, c-MYC [56].

**Figure 2:**
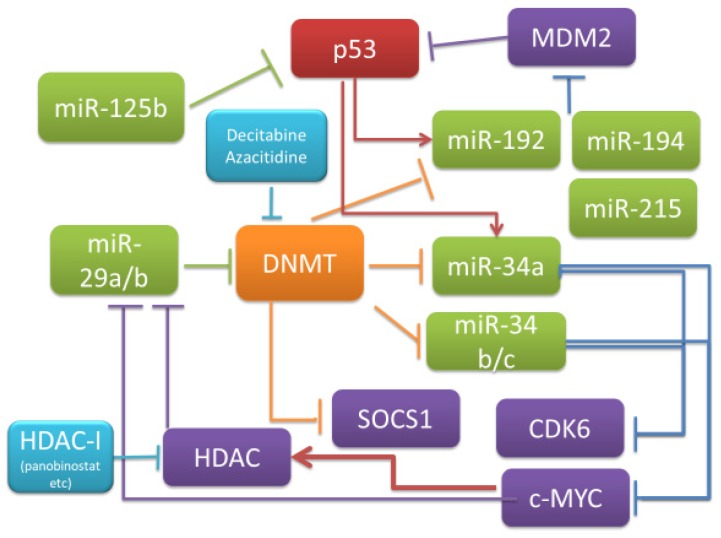
Feedback loop regulation network of microRNA and epigenetic modifier and potential drugs.

On the other hand, a subclass of miRNAs, named “epi-miRNAs”, that targets epigenetic regulators, such as DNA methyltransferases (DNMTs), HDACs, and components of PRC, have proven to contribute to the epigenetic cellular landscape in cancer [57]. Among a variety of miRNAs, the miR-29 family represents the prototypical example of epi-miRNAs, since miR-29s have been demonstrated to target a number of epigenetic effectors, thus inhibiting their aberrant expression and activity and leading to the re-activation of relevant oncosuppressive pathways in hematologic malignancies [58,59,60]. The 3’-UTR of the DNA methylating enzymes DNMT3A and 3B mRNA has a sequence complementary to miR-29a/b, and, in fact, it has been shown that the latter represses DNMT expression [61]. Expression of DNMT1, an enzyme that maintains the methylation status of methylated DNA, is regulated by the transcription factor SP1, and the expression of SP1 is also suppressed by miR-29b, suggesting that gene regulation by DNA methylation and miRNAs are mutually regulated [58,59]. miR-29b induces the expression of suppressor of cytokine signaling-1 (SOCS-1) by demethylation of its promoter [62]. c-MYC takes part in a multi-component regulatory complex that trans-represses several miRNAs in MM, including miR-23b, miR-29b, and miR-29a [63,64,65]. We also found that the expression level of DNMT was decreased and miR-34 expression was increased by the introduction of synthetic miR-29 in MM cells in vitro. *MYC*, which is a target of miR-34, was reported to be able to suppress miR-29b expression [47,48,63]. Altogether, these findings suggest a feedback loop regulation system in which miR-29 represses DNMT, which in turn is unable to exert its suppressing activity on miR-34, a miRNA that targets *MYC*, which controls the expression of miR-29 (Figure 2).

## 9. Other miRNA Regulatory Mechanisms in MM

Various factors are involved in the expression of mature miRNA: Drosha, which is involved in the production of pre-miRNAs; exportin-5, which transports pre-miRNAs out of the nucleus; and Dicer, which turns pre-miRNAs into mature miRNAs. Aberrant expression of Dicer, but not of Drosha, has been reported in MM [65]. Moreover, Dicer expression decreases with the progression from MGUS to MM, and progression-free survival (PFS) is prolonged when Dicer expression is high in MM [66]. This suggests that the decreased Dicer expression in MM attributes to decreased mature miRNA expression.

Emerging evidence suggests that miRNAs are themselves regulated by other RNA molecules that contain complementary miRNA binding sites, such as mRNAs, pseudogenes, lncRNAs, and circular RNAs (circRNAs). In recent years, endogenous non-coding RNAs, i.e., competing endogenous RNAs (ceRNA), have been discovered to enclose sequences complementary to miRNAs and to act as so-called sponges to suppress miRNA function. This is a key mechanism controlling miRNA function and has received considerable attention [67,68,69,70]. PTENP1, a pseudogene of the tumor suppressor gene PTEN, also has a region that binds to miR-21 and functions as ceRNA [70].

## 10. Drug Resistance and miRNA

Many studies have demonstrated that miRNAs might be involved in the drug resistance of MM (Table 2). Tumor suppressor miRNAs are frequently downregulated in drug-resistant MM cells, and restoring these miRNAs can overcome drug resistance through resensitization to chemotherapeutic agents [71]. For example, IL-6-suppressed miR-15a/16 expression induces drug resistance in MM cells and is associated with poor prognosis [72]. Three studies have identified miR-137, which targets the anti-apoptotic gene *MCL1*, *AURKA* (a gene coding for a protein involved in mitosis and cell proliferation), and AKT, as a tumor suppressing miRNA, and its overexpression in MM cells sensitizes them to anti-myeloma drugs [73,74,75]. Interestingly, in MM, miR-137 is silenced by promoter hypermethylation. Ectopic expression of miR-137 sensitized the cells to bortezomib via upregulating p53 and downregulating ATM/Chk2. miR-27a, miR-631, miR-324-5p, miR-155, miR-497, miR-520g, and miR-520h are shown to be involved in bortezomib resistance of MM cells, and inducing the expression of these miRNAs resensitizes the cells to bortezomib [31,76,77,78,79,80,81].

On the other hand, oncomiRs such as miR-21, miR-221/222, miR-125a, b, and miR-451 are upregulated in MM cells showing drug resistance. The enforced expression of these miRNAs reduced the cell death induced by anti-myeloma agents such as bortezomib and dexamethasone; inhibition of these miRNAs is thought to be a new therapeutic strategy to overcome drug resistance [19,20,21,22,39,42]. Gullà et al. and Zhao et al. demonstrated that miR-221/222 directly targets the BH3-only BCL-2 family member PUMA, which is a critical mediator of p53-dependent and -independent apoptosis. In vitro inhibition of miR-221/222 upregulated the expression of the pro-apoptotic genes *BAX* and *BAK*, and consequently abrogated dexamethasone resistance. Moreover, miR-221/222 expression inversely correlated with melphalan sensitivity of MM cells. Inhibition of miR-221/222 overcame melphalan resistance and triggered apoptosis of MM cells in vitro. Accordingly, treatment of SCID/NOD mice bearing melphalan-refractory human MM xenografts with systemic locked nucleic acid (LNA) inhibitors of miR-221 (LNA-i-miR-221) plus melphalan overcame drug resistance [20,21]. Furthermore, miR-221/222-mediated inhibition of autophagy was shown to promote dexamethasone resistance in MM [82]. miR-125a, b, and miR-221 inhibitors might be good candidates for the treatment of MM (Table 2).

## 11. Possible Classification of MM by miRNA Expression Profiling

With the development of miRNA microarrays, beads-based flow cytometry, and high-throughput deep sequencing, researchers are rapidly investigating how genome-wide miRNA profiles (miRNome) can aid in tumor classification, diagnosis, and prognosis prediction. miRNA profiles are thought to be more accurate than mRNA expression profiles because they not only distinguish normal from cancerous tissues and the organ or tissue from which cancer originates, but also distinguish subtypes of cancer in the same tissue. One study reported that 48 miRNAs accurately diagnosed 86% of cancers. Thus, the development of a miRNA-directed cancer classification is invaluable in clinical diagnosis and treatment planning. It has also been suggested that the expression pattern of miRNAs can be a prognostic biomarker.

Several miRNAs expressed in MM cells derived from patients were shown to be prognostic predictors. The miRNA-15a/16-1 cluster located in the chromosomal region 13q14 is downregulated but displays different expression patterns and prognostic significance in MM. Low miR-15a expression was a powerful independent predictor of progression free survival (PFS) and overall survival (OS) [83]; however, another study demonstrated opposite results [84]. A prognostic classification based on microarray-analyzed miRNA expression profiling has also been reported by the United Kingdom Medical Research Council (MRC). MM were classified into three groups according to the expression levels of miR-17 and miR-886-5p, and this method was found to be superior to the International Staging System (ISS) and fluorescence in situ hybridization (FISH)-based prognostic predictions [85]. The survival analysis based on miRNAs assessed by microarray reported by Chi et al. showed that low expression of miR-153, miR-490, miR-455, miR-642, miR-548d, miR-500, and miR-296, and high expression of miR-373, miR-554, and miR-888 were associated with poor prognosis [30]. A systemic review and meta-analysis were performed to confirm the prognostic significance of miRNA. Ten relevant studies including 1214 cases were analyzed, and it was revealed that upregulated miR-92a and downregulated miR-16, miR-25, miR-744, miR-15a, let-7e, and miR19b expression were significantly associated with poor prognosis of MM [86]. Given that miR-744 is located on chromosomal region 17p12, close to the *TP53* gene, and that deletions 17p13.1 to 17p12 are well-known indicators of poor prognosis, it is suggested that miR-744 might contribute to the poor prognosis of MM with deletion 17p [86]. miR-33b located in chromosome 17p is also significantly downregulated in MM, especially MM with high-risk cytogenetics, and the survival of patients with low miR-33b expression is significantly shorter [87]. Epigenetically suppressed miR-137 expression by promoter methylation is also associated with poor prognosis [75].

Because miRNAs are smaller and more stable than long RNAs, they can be more reliably extracted from frozen and paraffin-fixed tissues, plasma, serum, urine, saliva, and even sputum than mRNAs, suggesting that serum miRNAs from cancer patients are potential biomarkers [88]. Recent studies have also shown that miRNAs are not only present in cells, but also migrate in vivo in membrane-enclosed particles, called exosomes, being transported between cells [89,90]. Diagnosis and prognosis of MM, among others, are also expected to be obtained from the blood exome analysis [91].

## 12. Circulating miRNA in the Bloodstream

**Figure 3:**
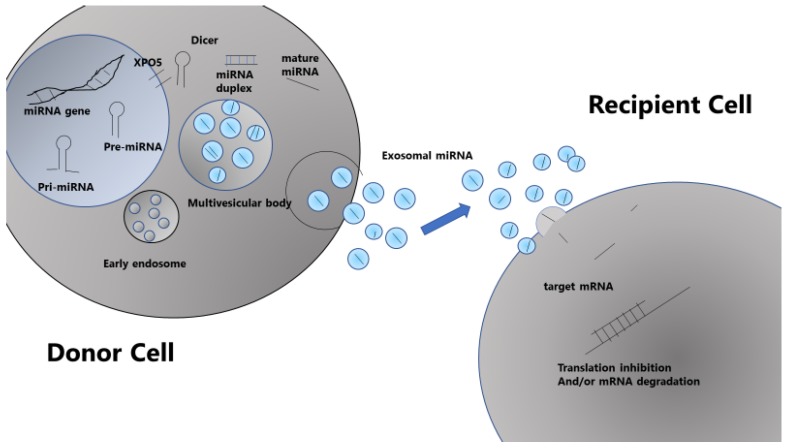
Intercellular communication via exosomes containing miRNAs [89].

Circulating miRNAs detected in body fluid have emerged as appealing biomarkers because they can be obtained by noninvasive methods and have been reported to be prognostic tools in many types of cancers including MM. miRNAs are detectable in body fluid encapsulated in lipid vesicles called extracellular vesicles (EVs) characterized according to the size into exosomes, microvesicles, and apoptotic bodies [89,90,91,92]. In addition to EVs, circulating miRNAs can be loaded into a high density lipoprotein (HDL) [93] or bound in a protein complex composed of an AGO family outside of vesicles [94]. Some secreted miRNAs, especially those in EVs such as exosomes, may mediate paracrine and endocrine communication between different tissues, thus modulating gene expression and function in distal cells [89,90] (Figure 3). Exosomes are 50–140 nm vesicles that contain protein and nucleic acids such as miRNAs. They are actively secreted by several cell types, including cancer cells, and can be isolated from peripheral blood, which makes them attractive biomarkers of disease progression and risk stratification [91]. In MM, bone marrow mesenchymal stromal cell-derived exosomes containing oncogenic proteins facilitate disease progression; interestingly, exosomes from MM patients contain the tumor suppressor miR-15a compared to those from healthy donors, which demonstrates the tumor-suppressive role of this miRNA [95].

The prognostic relevance of circulating exosomal miRNAs was demonstrated. When exosomes obtained from uniformly treated myeloma patients were analyzed, let-7b, let-7e, miR-106a, miR-106b, miR-155, miR-16, miR-17, miR-18a, and miR-20a were found to be significant risk factors for PFS. Among these, two miRNAs, let-7b and miR-18a, were significantly associated with both PFS and overall survival, even after adjustment for the ISS and adverse cytogenetics, supporting the use of circulating exosomal miRNAs to improve the identification of poor outcome of myeloma patients [96]. The let-7 family acts as tumor suppressor miRNAs in MM, with a low level of let-7 inducing cell proliferation and growth by depressing oncogenes such as *CCND1*, *MYC*, and *RAS*. miR-18a is a component of the miR-17-92 cluster in chromosomal region 13q31.3 and was reported to be associated with HIF-1 regulation and tumor metastasis.

In addition, not only survival risk factors, but also the different profile of exosomal miRNAs between smoldering MM and active MM, have been reported. The let-7 family members’ let-7c, miR-20a, miR-103a, miR-140, and miR-185a were significantly lower in MM, whereas miR-4505 and miR-4741 were found to be higher in this disease, suggesting that serum exosomal miRNAs can be used as biomarkers for disease progression [97]. Exosomal miRNAs can also be used as biomarkers for drug resistance, as a significant down-regulation of exosomal miR-16-5p and miR-15a-5, which target BCL-2, and miR-20a-5p and miR-17-5p, which target *MYC*, was revealed in bortezomib-resistant patients [98].

All these studies demonstrated the clinical usefulness of exosomal miRNAs as biomarkers. Employing exosomal miRNAs as biomarkers has a clear advantage compare to AGO-miRNA concerning their stability during the processing of genetic materials in circulation as well as functional role of exosomes in cell to cell communication [92]. However, whether these low contents of tumor-suppressive miRNAs in exosomes is just a marker of downregulation of these miRNAs in MM cells or plays some roles in promoting disease progression and drug resistance remains to be determined. Differential miRNA expression in exosomes may depend on selective miRNA sorting by four potential mechanisms: neural sphingomyelinase 2 dependent pathway, a mechanism involving a GGAG miRNA motif that can be recognized by sumoylated heterogeneous nuclear ribonucleoprotein, uridylation or adenylation of 30 ends of miRNA, or a mechanism which involves a miRNA induced silencing complex (miRISC) [92]. Since exosomes are known to be used as communication tools between cells by delivering their content, the putative role and mechanisms of exosomal miRNAs in this context should be clarified.

## 13. miRNA and the Microenvironment

The importance of the tumor microenvironment in cancer progression has been swiftly recognized. The cancer microenvironment comprises feeder cells such as fibroblasts, stromal cells, and mesenchymal stem cells (MSCs), immune cells such as lymphocytes (T, B, NK, among others), macrophages, and dendritic cells (DCs), and extracellular matrices, among others. Tumor cells and the cells in the tumor microenvironment communicate with each other through hormones and cytokines. Recently, EVs, including exosomes, containing miRNAs, RNAs, and proteins have emerged as important cellular communicators, and attention has been drawn to the functional role of miRNAs as well as their role as prognostic indicators.

Roccaro et al. showed that abnormal miRNA expression in MM was observed not only in MM cells, but also in stromal cells forming the bone marrow microenvironment, miRNAs in exosomes secreted by stromal cells derived from MM bone marrow were different from those secreted by normal stromal cells, and normal exosomes can suppress the proliferation of MM [95].

Bone marrow fibroblasts overexpress miR-27b and miR-214 in step with MM progression, which is dependent on tumor cell-derived exosomes. The specific miR profile in bone marrow fibroblasts parallels the transition from MGUS to MM. Overexpression of miR-27b-3p and miR-214-3p triggers proliferation and apoptosis resistance in myeloma fibroblasts via the FBXW7 and PTEN/AKT/GSK3 pathways, respectively. Transient transfection of miR-27b-3p and miR-214-3p inhibitors demonstrates cooperation between these two miRNAs in the expression of the anti-apoptotic factor MCL1, suggesting that miR-27b-3p and miR-214-3p negatively regulate myeloma fibroblast apoptosis. Furthermore, myeloma cells modulate miR-27b-3p and miR-214-3p expression in fibroblasts through the release of exosomes [99].

When miRNA changes were examined in MSCs after culture with conditioned medium for MM cells, 19 miRNAs were found to be dysregulated, including the upregulated miR-146a. Exosomes derived from MM cells contained miR-146a and could be transferred into MSCs. After overexpressing miR-146a in MSCs, the secretion of several cytokines and chemokines including CXCL1, IL-6, IL-8, IP-10, MCP-1, and CCL-5 was elevated, resulting in the enhancement of MM cell viability and migration [100].

DCs have a key role in regulating tumor immunity, tumor cell growth, and drug resistance. miR-29b was identified as the only miRNA upregulated in normal mature DCs and significantly downregulated in tumor-associated DCs. The enforced expression of miR-29b counteracted proinflammatory pathways, including STAT3 and NF-κB, and cytokine/chemokine signaling networks, which correlated with patients’ adverse prognosis and development of bone disease. MM reprograms the DC functional phenotype by downregulating miR-29b, whose reconstitution impairs the DCs ability to sustain MM cell growth and survival [101].

Emerging experimental evidence is also disclosing a key regulatory role of miRNAs in the regulation of bone homeostasis. The enforced expression of miR-29b impairs osteoclast differentiation and overcomes osteoclast activation triggered by MM cells [102]. Conversely, miR-138 expression is significantly increased in MSCs derived from MM and MM cells themselves, and inhibition of this miRNA enhances bone formation in the MM bone marrow niche [103].

## 14. Potential Therapies Using miRNA

The appeal and advantage of miRNAs as therapeutic agents lie in their ability to target genes of interest and to control various intracellular and intercellular networks [104].

miR-15a/16-1 was administered to in vivo xenograft models, resulting in tumor cell shrinkage [105]. Transient expression of miR-34a synthetic mimics or lentivirus-based miR-34a-stable enforced expression triggered growth inhibition and apoptosis of MM cells in vitro. Synthetic miR-34a downregulated the canonic targets BCL-2, CDK6, and NOTCH1 at both the mRNA and protein levels. Lentiviral vector-transduced MM xenografts with constitutive miR-34a expression showed high growth inhibition, and lipidic-formulated miR-34a showed an anti-MM activity in vivo [106]. Concerning the potential therapies using miR-34a, the combination with other anti-cancer agents appears a promising anti-MM strategy. For example, the combination of miR-34a and γ-secretase inhibitor, Sirtinol or zoledronic acid, as a new strategy to enhance the miR-34a-dependent inhibitory effect on its canonical targets [107].

The oncomiR miR-221/222 has shown to be a good candidate therapeutic target. miR-221/222 inhibitors triggered in vitro anti-proliferative effects and up-regulation of canonic miR-221/222 targets, including p27Kip1, PUMA, PTEN, and p57Kip2, in MM cells highly expressing miR-221/222. Conversely, transfection of miR-221/222 mimics increased the S-phase and downregulated p27Kip1 protein expression in MM with low basal miR-221/222 levels. The effects of miR-221/222 inhibitors were also evaluated in MM xenografts in SCID/ NOD mice [21,108].

Furthermore, LNA-i-miR-221 restored the drug sensitivity in melphalan-refractory MM cells. The inhibition of miR-221/222 overcame melphalan resistance and triggered apoptosis of MM cells in vitro, with in vivo experiments showing similar results [21]. miR-29b replacement has also been shown to inhibit proteasomes and to disrupt aggresome and autophagosome formation to enhance the anti-myeloma activity of bortezomib [109].

In order to use miRNAs as therapeutic agents, two problems must be solved: how to maintain their stability in vivo and how to develop delivery systems that efficiently deliver them to target cells. To date, methods using atelocollagen and liposome or viral vectors, such as adenoviruses, have been reported but a safe and reliable method has not yet been established. Given the stability of exosomes in the blood, treatment with exosomes containing tumor-suppressive miRNAs may also be considered. Cell therapy with stromal or mesenchymal cells that secrete exosomes containing tumor-suppressive miRNAs might also be effective.

However, conflicting findings do not support the classification of miRNAs as either oncogenic or tumor-suppressive. Certain miRNAs have been shown to be oncogenic in one scenario but tumor suppressive in another. For example, miR-125b acts as an oncomiR in the vast majority of hematologic malignancies and as a tumor suppressor in many solid tumors [12,110,111]. Therefore, therapeutic approaches using miRNAs should be considered with caution.

## 15. Conclusions

miRNAs are not junk and can serve as prognostic indicators and novel treatment targets. The ability of miRNAs to control the expression of many genes is attractive for the development of treatment strategies but one should be careful to use them, as their mechanism of regulation and function has not been yet clarified in depth. Thus, further studies are required to fully elucidate these mechanisms and to enable the safe use of miRNAs in the clinic.

## Figures and Tables

**Table 1:** Chromosomal abnormality and deregulated miRNA expression.

Cytogenetic Group	Deregulated miRNA	Chromosomal Location	miRNA Target	Ref.
t(4;14)	miR-133b	6p12.2	FSCN1	[29]
	miR-135b	1q32.1		
	miR-146a	5q34	IRAK1, Fas, SMAD4, TBP, CCL8/MCP2	
	miR-155	21q21.3	KPC1, IL-13Rα1, CYR61, SMAD1, SMAD2, SMAD5, HIVEP2, CEBPB, RUNX2, MYO10, JARID2, AGTR1	
	miR-193a	17q11.2		
	miR-196b	7p15.2		
	miR-203	14q32.33	P63, SOCS-3	
	miR-215	1q41	DHFR, TS	
	miR-342	14q32.2		
	miR-375	2q35	YAP, RASD1, PDK1, 14-3-3Zeta	
	miR-650	22q11.22	NDRG2, ING4	
t(11;14)	miR-95	4p16.1	SNX1	[29]
	miR-125a	19q13.41	PDPN, BAK1, KLF13, preproET1, ARID3B, HuR, ERBB2, ERBB3	
	miR-184	15q25.1	AKT2	
	miR-199a	19p13.2/1q24.3	CD44, mTOR, c-MET, HIF1-α	
	miR-215	1q41	DHFR, TS	
	miR-375	2q35	YAP, RASD1, PDK1, 14-3-3Zeta	
	miR-650	22q13.41	NDRG2, ING4	
t(14;16)	miR-1	20q13.33	TAGLN2, KLF4, c-MET	[25,29]
	miR-99b	19q13.41		
	miR-125a-5p	19q13.42	PDPN, BAK1, KLF13, preproET1, ARID3B, HuR, ERBB2, ERBB3	
	miR-133a	18q11.2/20q13.33		
	miR-135b	1q32.1		
	miR-196b	7p15.2		
	miR-214	1q24.3	PTEN	
	miR-375	2p35	YAP, RASD1, PDK1, 14-3-3Zeta	
	miR-642	19q13.32		
Deletion 13q14	miR-15a-16	13q14.3	E2F, CCND1, WNT3A, BCL-2	[16,27]
	miR-181a/b	1q32.1/9q33.3	RASSF1A, TIMP3, NLK, Prox1, HOXA11	
	miR-221	Xp11.3	p27, ETS1, PUMA, p57, TIMP3, PTEN	
	miR-222	Xp11.3	p27, PUMA, p57, TIMP3, PETN	
	miR-382	14q32.31	SOD2, NPM1, PSPC1, HSPD1, ECH1	
1q gain	miR-205	1q32.2		[25]
	miR-215	1q41	MDM2, RUNX1	
	miR-488	1q25.2		
	miR-1231	1q32.1		
Deletion 17p	miR-22	17p13.3		[25,29]
	miR-324-5p	17p13.1	MDR, MRP, BCRP, BCL-2 family	[31]

**Table 2:** miRNAs and their potential role in multiple myeloma (MM) drug resistance.

miRNA	Observed Alteration	Target	Functional Response	Ref
15a/16	Downregulated	NA	IL-6 downregulates miR-15a/16 and enhances drug resistance	[72]
221/222	Upregulated in melphalan-resistant HMCLs	PUMA/BBC3	miR-221/222 inhibitor upregulates PUMA, increasing apoptosis in drug-resistant HMCLs	[20,21]
221/222	Upregulated in DEX-resistant HMCL (MM.1R)	PUMA/BBC3	Inhibition of miR-221/222 in MM.1R cells partially restores their DEX sensitivity, whereas enforced expression in MM.1S cells downregulates PUMA and renders them resistant to DEX	[22]
125a	Upregulated in MM cells following adhesion to BMSCs	p53	NA	[39]
125b	Upregulated in DEX-responsive MM cells	p53, interacts with miR-34a targeting SIRT1	Anti-miR-125b increases p53, miR-34a, decreased SIRT1, and increases DEX-induced apoptosis	[42]
137	Downregulated in MM cells harboring 1p12-21 deletion	MCL-1, AKT, AURKA	Targets *MCL1*, *AURKA*, and AKT; Ectopic expression of miR-137 sensitizes cells to bortezomib via upregulating p53 and downregulating ATM/Chk2	[73,74,75]
27a	Downregulated in BTZ-resistant HMCLs	CDK5	Ectopic expression of miR-27a in MM cells increases their sensitivity to BTZ	[76]
631	Downregulated in BTZ-resistant HMCL	UbcH10/MDR1	Modulates UbcH10/MDR1 pathway, which is associated with BTZ resistance in HMCL	[77]
324-5p	Located on 17p and downregulated in MM cells harboring 17p deletion	BCL2 family gene and MDR1, BCRP, MRP	Regulates sensitivity to bortezomib in MM cells by targeting hedgehog signaling	[31]
155	Downregulated in MM cells	PSMβ5	miR-155 elicits anti-MM activity likely via proteasome inhibition	[78]
497	Downregulated in MM cells	BCL-2	miR-497 suppresses MM cell proliferation and promotes apoptosis by directly targeting BCL-2 and increases the sensitivity of MM cells to bortezomib	[79]
520g/520h	Downregulated in BTZ-resistant HMCL	APE1	Combined overexpression of miR-520g and miR-520h overcomes bortezomib resistance in MM through inhibition of DNA repair	[80]
21	Upregulated in MM cells following adhesion to BMSCs	RhoB	Enforced expression of miR-21 leads to reduced apoptosis induced by DEX, DOX, and BTZ; inhibition of this miRNA induces the opposite effects	[81]
21	Upregulated in melphalan-resistant HMCLs	NA	NA	[19]

NA: not available, HMCL: human myeloma cell line, DEX: dexamethasone, BMSC: bone marrow stroma cell, BTZ: bortezomib, DOX: doxorubicin.

## References

[1] Lee R.C., Feinbaum R.L., Ambros V. (1993). The *C. elegans* heterochronic gene lin-4 encodes small RNAs with antisense complementarity to lin-14. Cell.

[2] Reinhart B.J., Slack F.J., Basson M., Pasquinelli A.E., Bettinger J.C., Rougvie A.E., Horvitz H.R., Ruvkun G. (2000). The 21-nucleotide let-7 RNA regulates developmental timing in *Caenorhabditis elegans*. Nature.

[3] Berezikov E. (2011). Evolution of microRNA diversity and regulation in animals. Nat. Rev. Genet..

[4] Deline B., Greenwood J.M., Clark J.W., Puttick M.N., Peterson K.J., Donoghue P.C.J. (2018). Evolution of metazoan morphological disparity. Proc. Natl. Acad. Sci. USA.

[5] Tarver J.E., Donoghue P.C., Peterson K.J. (2012). Do miRNAs have a deep evolutionary history?. Bioessays.

[6] Romero-Cordoba S.L., Salido-Guadarrama I., Rodriguez-Dorantes M., Hidalgo-Miranda A. (2014). miRNA biogenesis: Biological impact in the development of cancer. Cancer Biol. Ther..

[7] Iorio M.V., Croce C.M. (2012). MicroRNA dysregulation in cancer: Diagnostics, monitoring and therapeutics. A comprehensive review. EMBO Mol. Med..

[8] Iorio M.V., Croce C.M. (2012). microRNA involvement in human cancer. Carcinogenesis.

[9] Aqeilan R.I., Calin G.A., Croce C.M. (2010). miR-15a and miR-16-1 in cancer: Discovery, function, and future perspectives. Cell Death Differ..

[10] Calin G.A., Dumitru C.D., Shimizu M., Bichi R., Zupo S., Noch E., Aldler H., Rattan S., Keating M., Rai K. (2002). Frequent deletions and down-regulation of micro-RNA genes miR15 and miR16 at 13q14 in chronic lymphocytic leukemia. Proc. Natl. Acad. Sci. USA.

[11] Cimmino A., Calin G.A., Fabbri M., Iorio M.V., Ferracin M., Shimizu M., Wojcik S.E., Aqeilan R.I., Zupo S., Dono M. (2005). miR-15 and miR-16 induce apoptosis by targeting BCL2. Proc. Natl. Acad. Sci. USA.

[12] Svoronos A.A., Engelman D.M., Slack F.J. (2016). OncomiR or Tumor Suppressor? The Duplicity of MicroRNAs in Cancer. Cancer Res..

[13] Al-Masri A., Price-Troska T., Chesi M., Chung T.H., Kim S., Carpten J., Bergsagel P.L., Fonseca R. (2005). MicroRNA Expression Analysis in Multiple Myeloma. Blood.

[14] Pichiorri F., Suh S.S., Ladetto M., Kuehl M., Palumbo T., Drandi D., Taccioli C., Zanesi N., Alder H., Hagan J.P. (2008). MicroRNAs regulate critical genes associated with multiple myeloma pathogenesis. Proc. Natl. Acad. Sci. USA.

[15] Lerner M., Harada M., Lovén J., Castro J., Davis Z., Oscier D., Henriksson M., Sangfelt O., Grandér D., Corcoran M.M. (2009). DLEU2, frequently deleted in malignancy, functions as a critical host gene of the cell cycle inhibitory microRNAs miR-15a and miR-16-1. Exp. Cell Res..

[16] Roccaro A.M., Sacco A., Thompson B., Leleu X., Azab A.K., Azab F., Runnels J., Jia X., Ngo H.T., Melhem M.R. (2009). MicroRNAs 15a and 16 regulate tumor proliferation in multiple myeloma. Blood.

[17] Handa H., Hattori H., Takahashi N., Sasaki Y., Saitoh T., Osaki Y., Tahara K., Koiso H., Mitsui T., Shimizu H. (2012). Association between micro-RNA and epigenetic modifiers DNA methyltransferases (DNMTs), histone deacetylases (HDACs) in multiple myeloma (MM) and monoclonal gammopathy with undetermined significance (MGUS). Blood.

[18] Löffler D., Brocke-Heidrich K., Pfeifer G., Stocsits C., Hackermüller J., Kretzschmar A.K., Burger R., Gramatzki M., Blumert C., Bauer K. (2007). Interleukin-6 dependent survival of multiple myeloma cells involves the Stat3-mediated induction of microRNA-21 through a highly conserved enhancer. Blood.

[19] Leone E., Morelli E., Di Martino M.T., Amodio N., Foresta U., Gullà A., Rossi M., Neri A., Giordano A., Munshi N.C. (2013). Targeting miR-21 inhibits in vitro and in vivo multiple myeloma cell growth. Clin. Cancer Res..

[20] Di Martino M.T., Gullà A., Cantafio M.E., Lionetti M., Leone E., Amodio N., Guzzi P.H., Foresta U., Conforti F., Cannataro M. (2013). In vitro and in vivo anti-tumor activity of miR-221/222 inhibitors in multiple myeloma. Oncotarget.

[21] Gullà A., Di Martino M.T., Gallo Cantafio M.E., Morelli E., Amodio N., Botta C., Pitari M.R., Lio S.G., Britti D., Stamato M.A. (2016). A 13 mer LNA-i-miR-221 inhibitor restores drug sensitivity in melphalan-refractory multiple myeloma cells. Clin. Cancer Res..

[22] Zhao J.J., Chu Z.B., Hu Y., Lin J., Wang Z., Jiang M., Chen M., Wang X., Kang Y., Zhou Y. (2015). Targeting the miR-221/222/PUMA/BAK/BAX pathway abrogates dexamethasone resistance in multiple myeloma. Cancer Res..

[23] Qu X.Y., Zhang S.S., Wu S., Hong M., Li J.Y., Chen L.J., Xu J.R. (2013). Expression level of microRNA-92a and its clinical significance in multiple myeloma patients. Chin. J. Hematol..

[24] Zhang Y.K., Wang H., Leng Y., Li Z.L., Yang Y.F., Xiao F.J., Li Q.F., Chen X.Q., Wang L.S. (2011). Overexpression of microRNA-29b induces apoptosis of multiple myeloma cells through down regulating Mcl-1. Biochem. Biophys. Res. Commun..

[25] Lionetti M., Biasiolo M., Agnelli L., Todoerti K., Mosca L., Fabris S., Sales G., Deliliers G.L., Bicciato S., Lombardi L. (2009). Identification of microRNA expression patterns and definition of a microRNA/mRNA regulatory network in distinct molecular groups of multiple myeloma. Blood.

[26] Corthals S.L., Jongen-Lavrencic M., de Knegt Y., Peeters J.K., Beverloo H.B., Lokhorst H.M., Sonneveld P. (2010). Micro-RNA-15a and micro-RNA-16 expression and chromosome 13 deletions in multiple myeloma. Leuk. Res..

[27] Corthals S.L., Sun S.M., Kuiper R., de Knegt Y., Broyl A., van der Holt B., Beverloo H.B., Peeters J.K., el Jarari L., Lokhorst H.M. (2011). MicroRNA signatures characterize multiple myeloma patients. Leukemia.

[28] Lionetti M., Agnelli L., Mosca L., Fabris S., Andronache A., Todoerti K., Ronchetti D., Deliliers G.L., Neri A. (2009). Integrative high-resolution microarray analysis of human myeloma cell lines reveals deregulated miRNA expression associated with allelic imbalances and gene expression profiles. Genes Chromosomes Cancer.

[29] Gutiérrez N.C., Sarasquete M.E., Misiewicz-Krzeminska I., Delgado M., De Las Rivas J., Ticona F.V., Fermiñán E., Martín-Jiménez P., Chillón C., Risueño A. (2010). Deregulation of microRNA expression in the different genetic subtypes of multiple myeloma and correlation with gene expression profiling. Leukemia.

[30] Chi J., Ballabio E., Chen X.H., Kušec R., Taylor S., Hay D., Tramonti D., Saunders N.J., Littlewood T., Pezzella F. (2011). MicroRNA expression in multiple myeloma is associated with genetic subtype, isotype and survival. Biol. Direct.

[31] Tang B., Xu A., Xu J., Huang H., Chen L., Su Y., Zhang L., Li J., Fan F., Deng J. (2018). MicroRNA-324-5p regulates stemness, pathogenesis and sensitivity to bortezomib in multiple myeloma cells by targeting hedgehog signaling. Int. J. Cancer.

[32] Boyd K.D., Ross F.M., Tapper W.J., Chiecchio L., Dagrada G., Konn Z.J., Gonzalez D., Walker B.A., Hockley S.L., Wardell C.P. (2011). NCRI Haematology Oncology Studies Group. The clinical impact and molecular biology of del(17p) in multiple myeloma treated with conventional or thalidomide-based therapy. Genes Chromosomes Cancer.

[33] Avet-Loiseau H., Attal M., Campion L., Caillot D., Hulin C., Marit G., Stoppa A.M., Voillat L., Wetterwald M., Pegourie B. (2012). Long-term analysis of the IFM 99 trials for myeloma: Cytogenetic abnormalities [t(4;14), del(17p), 1q gains] play a major role in defining long-term survival. J. Clin. Oncol..

[34] Avet-Loiseau H., Durie B.G., Cavo M., Attal M., Gutierrez N., Haessler J., Goldschmidt H., Hajek R., Lee J.H., Sezer O. (2013). International Myeloma Working Group. Combining fluorescent in situ hybridization data with ISS staging improves risk assessment in myeloma: An International Myeloma Working Group collaborative project. Leukemia.

[35] Nag S., Zhang X., Srivenugopal K.S., Wang M.H., Wang W., Zhang R. (2014). Targeting MDM2-p53 interaction for cancer therapy: Are we there yet?. Curr. Med. Chem..

[36] Elnenaei M.O., Gruszka-Westwood A.M., A’Hernt R., Matutes E., Sirohi B., Powles R., Catovsky D. (2003). Gene abnormalities in multiple myeloma; the relevance of TP53, MDM2, and CDKN2A. Haematologica.

[37] Pichiorri F., Suh S.S., Rocci A., De Luca L., Taccioli C., Santhanam R., Zhou W., Benson D.M., Hofmainster C., Alder H. (2010). Downregulation of p53-inducible microRNAs 192, 194, and 215 impairs the p53/MDM2 autoregulatory loop in multiple myeloma development. Cancer Cell.

[38] Hünten S., Siemens H., Kaller M., Hermeking H. (2013). The p53/microRNA network in cancer: Experimental and bioinformatics approaches. Adv. Exp. Med. Biol..

[39] Leotta M., Biamonte L., Raimondi L., Ronchetti D., Di Martino M.T., Botta C., Leone E., Pitari M.R., Neri A., Giordano A. (2014). A p53-dependent tumor suppressor network is induced by selective miR-125a-5p inhibition in multiple myeloma cells. J. Cell. Physiol..

[40] Le M.T., Teh C., Shyh-Chang N., Xie H., Zhou B., Korzh V., Lodish H.F., Lim B. (2009). MicroRNA-125b is a novel negative regulator of p53. Genes Dev..

[41] Hu W., Chan C.S., Wu R., Zhang C., Sun Y., Song J.S., Tang L.H., Levine A.J., Feng Z. (2010). Negative regulation of tumor suppressor p53 by microRNA miR-504. Mol. Cell.

[42] Murray M.Y., Rushworth S.A., Zaitseva L., Bowles K.M., Macewan D.J. (2013). Attenuation of dexamethasone-induced cell death in multiple myeloma is mediated by miR-125b expression. Cell Cycle.

[43] Misso G., Zarone M.R., Lombardi A., Grimaldi A., Cossu A.M., Ferri C., Russo M., Vuoso D.C., Luce A., Kawasaki H. (2019). miR-125b Upregulates miR-34a and Sequentially Activates Stress Adaption and Cell Death Mechanisms in Multiple Myeloma. Mol. Ther. Nucleic Acids.

[44] Hermeking H. (2010). The miR-3a family in cancer and apoptosis. Cell Death Differ..

[45] Okada N., Lin C.P., Ribeiro M.C., Biton A., Lai G., He X., Bu P., Vogel H., Jablons D.M., Keller A.C. (2014). A positive feedback between p53 and miR-34 miRNAs mediates tumor suppression. Genes Dev..

[46] Chim C.S., Wong K.Y., Qi Y., Loong F., Lam W.L., Wong L.G., Jin D.Y., Costello J.F., Liang R. (2010). Epigenetic inactivation of the miR-34a in hematological malignancies. Carcinogenesis.

[47] Kimura K., Kuroda Y., Masuda Y., Yamane A., Hattori H., Tahara K., Kaneko A., Suda I., Takahashi N., Gotoh N. (2015). Loop regulation between microRNAs and epigenetics underlie microRNA dysregulation in multiple myeloma and is associated with the disease progression. Blood.

[48] Handa H. (2015). Aberrant micro RNA and epigenetic network are associated with progression from MGUS to multiple myeloma. Rinsho Ketsueki.

[49] Wong K.Y., Yim R.L., So C.C., Jin D.Y., Liang R., Chim C.S. (2011). Epigenetic inactivation of the MIR34B/C in multiple myeloma. Blood.

[50] Wong K.Y., Huang X., Chim C.S. (2012). DNA methylation of microRNA genes in multiple myeloma. Carcinogenesis.

[51] Zhang W., Wang Y.E., Zhang Y., Leleu X., Reagan M., Zhang Y., Mishima Y., Glavey S., Manier S., Sacco A. (2014). Global epigenetic regulation of microRNAs in multiple myeloma. PLoS ONE.

[52] Misiewicz-Krzeminska I., Krzeminski P., Corchete L.A., Quwaider D., Rojas E.A., Herrero A.B., Gutiérrez N.C. (2019). Factors regulating microRNA expression and function in multiple myeloma. Noncoding RNA.

[53] Tatekawa S., Chinen Y., Ri M., Narita T., Shimura Y., Matsumura-Kimoto Y., Tsukamoto T., Kobayashi T., Kawata E., Uoshima N. (2017). Epigenetic repression of miR-375 is the dominant mechanism for constitutive activation of the PDPK1/RPS6KA3 signalling axis in multiple myeloma. Br. J. Haematol..

[54] Amodio N., Stamato M.A., Gullà A.M., Morelli E., Romeo E., Raimondi L., Pitari M.R., Ferrandino I., Misso G., Caraglia M. (2016). Therapeutic Targeting of miR-29b/HDAC4 Epigenetic Loop in Multiple Myeloma. Mol. Cancer Ther..

[55] Stamato M.A., Juli G., Romeo E., Ronchetti D., Arbitrio M., Caracciolo D., Neri A., Tagliaferri P., Tassone P., Amodio N. (2017). Inhibition of EZH2 triggers the tumor suppressive miR-29b network in multiple myeloma. Oncotarget.

[56] Min D.J., Ezponda T., Kim M.K., Will C.M., Martinez-Garcia E., Popovic R., Basrur V., Elenitoba-Johnson K.S., Licht J.D. (2013). MMSET stimulates myeloma cell growth through microRNA-mediated modulation of c-MYC. Leukemia.

[57] Sandoval J., Esteller M. (2012). Cancer epigenomics: Beyond genomics. Curr. Opin. Genet. Dev..

[58] Amodio N., Leotta M., Bellizzi D., Di Martino M.T., D’Aquila P., Lionetti M., Fabiani F., Leone E., Gullà A.M., Passarino G. (2012). DNA-demethylating and anti-tumor activity of synthetic miR-29b mimics in multiple myeloma. Oncotarget.

[59] Garzon R., Liu S., Fabbri M., Liu Z., Heaphy C.E., Callegari E., Schwind S., Pang J., Yu J., Muthusamy N. (2009). MicroRNA-29b induces global DNA hypomethylation and tumor suppressor gene reexpression in acute myeloid leukemia by targeting directly DNMT3A and 3B and indirectly DNMT1. Blood.

[60] Liu S., Wu L.C., Pang J., Santhanam R., Schwind S., Wu Y.Z., Hickey C.J., Yu J., Becker H., Maharry K. (2010). Sp1/NFkappaB/HDAC/miR-29b regulatory network in KIT-driven myeloid leukemia. Cancer Cell.

[61] Fabbri M., Garzon R., Cimmino A., Liu Z., Zanesi N., Callegari E., Liu S., Alder H., Costinean S., Fernandez-Cymering C. (2007). MicroRNA-29 family reverts aberrant methylation in lung cancer by targeting DNA methyltransferases 3A and 3B. Proc. Natl. Acad. Sci. USA.

[62] Amodio N., Bellizzi D., Leotta M., Raimondi L., Biamonte L., D’Aquila P., Di Martino M.T., Calimeri T., Rossi M., Lionetti M. (2013). miR-29b induces SOCS-1 epression by promoter demethylation and negatively regulates migration of multiple myeloma and endothelial cells. Cell Cycle.

[63] Mott J.L., Kurita S., Cazanave S.C., Bronk S.F., Werneburg N.W., Fernandez-Zapico M.E. (2010). Transcriptional suppression of mir-29b-1/mir-29a promoter by c-Myc, hedgehog, and NF-kappaB. J. Cell. Biochem..

[64] Amodio N., Di Martino M.T., Foresta U., Leone E., Lionetti M., Leotta M., Gullà A.M., Pitari M.R., Conforti F., Rossi M. (2012). miR-29b sensitizes multiple myeloma cells to bortezomib-induced apoptosis through the activation of a feedback loop with the transcription factor Sp1. Cell Death Dis..

[65] Fulciniti M., Amodio N., Bandi R.L., Cagnetta A., Samur M.K., Acharya C., Prabhala R., D’Aquila P., Bellizzi D., Passarino G. (2016). miR-23b/SP1/c-myc forms a feed-forward loop supporting multiple myeloma cell growth. Blood Cancer J..

[66] Sarasquete M.E., Gutiérrez N.C., Misiewicz-Krzeminska I., Paiva B., Chillón M.C., Alcoceba M., García-Sanz R., Hernández J.M., González M., San-Miguel J.F. (2011). Upregulation of Dicer is more frequent in monoclonal gammopathies of undetermined significance than in multiple myeloma patients and is associated with longer survival in symptomatic myeloma patients. Haematologica.

[67] Cesana M., Cacchiarelli D., Legnini I., Santini T., Sthandier O., Chinappi M., Tramontano A., Bozzoni I. (2011). A long noncoding RNA controls muscle differentiation by functioning as a competing endogenous RNA. Cell.

[68] Salmena L., Poliseno L., Tay Y., Kats L., Pandolfi P.P. (2011). A ceRNA hypothesis: The Rosetta Stone of a hidden RNA language?. Cell.

[69] Thomson D.W., Dinger M.E. (2016). Endogenous microRNA sponges: Evidence and controversy. Nat. Rev. Genet..

[70] Yu G., Yao W., Gumireddy K., Li A., Wang J., Xiao W., Chen K., Xiao H., Li H., Tang K. (2014). Pseudogene PTENP1 functions as a competing endogenous RNA to suppress clear-cell renal cell carcinoma progression. Mol. Cancer Ther..

[71] Abdi J., Jian H., Chang H. (2016). Role of micro-RNAs in drug resistance of multiple myeloma. Oncotarget.

[72] Hao M., Zhang L., An G., Sui W., Yu Z., Zou D., Xu Y., Chang H., Qiu L. (2011). Suppressing miRNA-15a/-16 expression by interleukin-6 enhances drug-resistance in myeloma cells. J. Hematol. Oncol..

[73] Yang Y., Li F., Saha M.N., Abdi J., Qiu L., Chang H. (2015). miR-137 and miR-197 induce apoptosis and suppress tumorigenicity by targeting MCL-1 in multiple myeloma. Clin. Cancer Res..

[74] Zhang B., Ma L., Wei J., Hu J., Zhao Z., Wang Y., Chen Y., Zhao F. (2016). miR-137 suppresses the phosphorylation of AKT and improves the dexamethasone sensitivity in multiple myeloma cells via targeting MITF. Curr. Cancer Drug Targets.

[75] Qin Y., Zhang S., Deng S., An G., Qin X., Li F., Xu Y., Hao M., Yang Y., Zhou W. (2017). Epigenetic silencing of miR-137 induces drug resistance and chromosomal instability by targeting AURKA in multiple myeloma. Leukemia.

[76] Ballabio E., Armesto M., Breeze C.E., Manterola L., Arestin M., Tramonti D., Hatton C.S., Lawrie C.H. (2012). Bortezomib action in multiple myeloma: microRNA-mediated synergy (and miR-27a/CDK5 driven sensitivity)?. Blood Cancer J..

[77] Xi H., Li L., Du J., An R., Fan R., Lu J., Wu Y.X., Wu S.X., Hou J., Zhao L.M. (2017). hsa-miR-631 resensitizes bortezomib-resistant multiple myeloma cell lines by inhibiting UbcH10. Oncol. Rep..

[78] Amodio N., Gallo Cantafio M.E., Botta C., Agosti V., Federico C., Caracciolo D., Ronchetti D., Rossi M., Driessen C., Neri A. (2019). Replacement of miR-155 Elicits Tumor Suppressive Activity and Antagonizes Bortezomib Resistance in Multiple Myeloma. Cancers.

[79] Tian F., Zhan Y., Zhu W., Li J., Tang M., Chen X., Jiang J. (2019). MicroRNA-497 inhibits multiple myeloma growth and increases susceptibility to bortezomib by targeting Bcl-2. Int. J. Mol. Med..

[80] Yuan X., Ma R., Yang S., Jiang L., Wang Z., Zhu Z., Li H. (2019). miR-520g and miR-520h overcome bortezomib resistance in multiple myeloma via suppressing APE1. Cell Cycle.

[81] Wang X., Li C., Ju S., Wang Y., Wang H., Zhong R. (2011). Myeloma cell adhesion to bone marrow stromal cells confers drug resistance by microRNA-21 up-regulation. Leuk. Lymphoma.

[82] Xu J., Su Y., Xu A., Fan F., Mu S., Chen L., Chu Z., Zhang B., Huang H., Zhang J. (2019). miR-221/222-Mediated Inhibition of Autophagy Promotes Dexamethasone Resistance in Multiple Myeloma. Mol. Ther..

[83] Li F., Xu Y., Deng S., Li Z., Zou D., Yi S., Sui W., Hao M., Qiu L. (2015). MicroRNA-15a/16-1 cluster located at chromosome 13q14 is down-regulated but displays different expression pattern and prognostic significance in multiple myeloma. Oncotarget.

[84] Gao X., Zhang R., Qu X., Zhao M., Zhang S., Wu H., Jianyong L., Chen L. (2012). MiR-15a, miR-16-1 and miR-17-92 cluster expression are linked to poor prognosis in multiple myeloma. Leuk. Res..

[85] Wu P., Agnelli L., Walker B.A., Todoerti K., Lionetti M., Johnson D.C., Kaiser M., Mirabella F., Wardell C., Gregory W.M. (2013). Improved risk stratification in myeloma using a microRNA-based classifier. Br. J. Haematol..

[86] Xu P., Xia T., Ling Y., Chen B. (2019). MiRNAs with prognostic significance in multiple myeloma: A systemic review and meta-analysis. Medicine.

[87] Li F., Hao M., Feng X., Zang M., Qin Y., Yi S., Li Z., Xu Y., Zhou L., Sui W. (2015). Downregulated miR-33b is a novel predictor associated with disease progression and poor prognosis in multiple myeloma. Leuk. Res..

[88] Di Leva G., Croce C.M. (2013). miRNA profiling of cancer. Curr. Opin. Genet. Dev..

[89] Hannafon B.N., Ding W.Q. (2013). Intercellular Communication by Exosome-Derived microRNAs in Cancer. Int. J. Mol. Sci..

[90] Mori M.A., Ludwig R.G., Garcia-Martin R., Brandão B.B., Kahn C.R. (2019). Extracellular miRNAs: From Biomarkers to Mediators of Physiology and Disease. Cell Metab..

[91] Moloudizargari M., Abdollahi M., Asghari M.H., Zimta A.A., Neagoe I.B., Nabavi S.M. (2019). The emerging role of exosomes in multiple myeloma. Blood Rev..

[92] Grimaldi A., Zarone M.R., Irace C., Zappavigna S., Lombardi A., Kawasaki H., Caraglia M., Misso G. (2018). Non-coding RNAs as a new dawn in tumor diagnosis. Semin. Cell Dev. Biol..

[93] Vickers K.C., Palmisano B.T., Shoucri B.M., Shamburek R.D., Remaley A.T. (2011). MicroRNAs are transported in plasma and delivered to recipient cells by high-density lipoproteins. Nat. Cell Biol..

[94] Arroyo J.D., Chevillet J.R., Kroh E.M., Ruf I.K., Pritchard C.C., Gibson D.F., Mitchell P.S., Bennett C.F., Pogosova-Agadjanyan E.L., Stirewalt D.L. (2011). Argonaute2 complexes carry a population of circulating microRNAs independent of vesicles in human plasma. Proc. Natl. Acad. Sci. USA.

[95] Roccaro A.M., Sacco A., Maiso P., Azab A.K., Tai Y.T., Reagan M., Azab F., Flores L.M., Campigotto F., Weller E. (2013). BM mesenchymal stromal cell-derived exosomes facilitate multiple myeloma progression. J. Clin. Investig..

[96] Manier S., Liu C.J., Avet-Loiseau H., Park J., Shi J., Campigotto F., Salem K.Z., Huynh D., Glavey S.V., Rivotto B. (2017). Prognostic role of circulating exosomal miRNAs in multiple myeloma. Blood.

[97] Zhang Z.Y., Li Y.C., Geng C.Y., Zhou H.X., Gao W., Chen W.M. (2019). Serum exosomal microRNAs as novel biomarkers for multiple myeloma. Hematol. Oncol..

[98] Zhang L., Pan L., Xiang B., Zhu H., Wu Y., Chen M., Guan P., Zou X., Valencia C.A., Dong B. (2016). Potential role of exosome-associated microRNA panels and in vivo environment to predict drug resistance for patients with multiple myeloma. Oncotarget.

[99] Frassanito M.A., Desantis V., Di Marzo L., Craparotta I., Beltrame L., Marchini S., Annese T., Visino F., Arciuli M., Saltarella I. (2019). Bone marrow fibroblasts overexpress miR-27b and miR-214 in step with multiple myeloma progression, dependent on tumour cell-derived exosomes. J. Pathol..

[100] De Veirman K., Wang J., Xu S., Leleu X., Himpe E., Maes K., De Bruyne E., Van Valckenborgh E., Vanderkerken K., Menu E. (2016). Induction of miR-146a by multiple myeloma cells in mesenchymal stromal cells stimulates their pro-tumoral activity. Cancer Lett..

[101] Botta C., Cucè M., Pitari M.R., Caracciolo D., Gullà A., Morelli E., Riillo C., Biamonte L., Gallo Cantafio M.E., Prabhala R. (2018). MiR-29b antagonizes the pro-inflammatory tumor-promoting activity of multiple myeloma-educated dendritic cells. Leukemia.

[102] Rossi M., Pitari M.R., Amodio N., Di Martino M.T., Conforti F., Leone E., Botta C., Paolino F.M., Del Giudice T., Iuliano E. (2013). miR-29b negatively regulates human osteoclastic cell differentiation and function: Implications for the treatment of multiple myeloma-related bone disease. J. Cell. Physiol..

[103] Tsukamoto S., Løvendorf M.B., Park J., Salem K.Z., Reagan M.R., Manier S., Zavidij O., Rahmat M., Huynh D., Takagi S. (2018). Inhibition of microRNA-138 enhances bone formation in multiple myeloma bone marrow niche. Leukemia.

[104] Garzon R., Marcucci G., Croce C.M. (2010). Targeting microRNAs in cancer: Rationale, strategies and challenges. Nat. Rev. Drug Discov..

[105] Sun C.Y., She X.M., Qin Y., Chu Z.B., Chen L., Ai L.S., Zhang L., Hu Y. (2013). miR-15a and miR-16 affect the angiogenesis of multiple myeloma by targeting VEGF. Carcinogenesis.

[106] Di Martino M.T., Leone E., Amodio N., Foresta U., Lionetti M., Pitari M.R., Cantafio M.E., Gullà A., Conforti F., Morelli E. (2012). Synthetic miR-34a mimics as a novel therapeutic agent for multiple myeloma: In vitro and in vivo evidence. Clin. Cancer Res..

[107] Zarone M.R., Misso G., Grimaldi A., Zappavigna S., Russo M., Amler E., Di Martino M.T., Amodio N., Tagliaferri P., Tassone P. (2017). Evidence of novel miR-34a-based therapeutic approaches for multiple myeloma treatment. Sci. Rep..

[108] Di Martino M.T., Gullà A., Gallo Cantafio M.E., Altomare E., Amodio N., Leone E., Morelli E., Lio S.G., Caracciolo D., Rossi M. (2014). In vitro and in vivo activity of a novel locked nucleic acid (LNA)-inhibitor-miR-221 against multiple myeloma cells. PLoS ONE.

[109] Jagannathan S., Vad N., Vallabhapurapu S., Vallabhapurapu S., Anderson K.C., Driscoll J.J. (2015). MiR-29b replacement inhibits proteasomes and disrupts aggresome+autophagosome formation to enhance the antimyeloma benefit of bortezomib. Leukemia.

[110] Shaham L., Binder V., Gefen N., Borkhardt A., Izraeli S. (2012). MiR-125 in normal and malignant hematopoiesis. Leukemia.

[111] Sun Y.M., Lin K.Y., Chen Y.Q. (2013). Diverse functions of miR-125 family in different cell contexts. J. Hematol. Oncol..

